# Correlation Analysis of Nodular Sonographic Parameters with Cervical Lymph Node Metastases in Papillary Thyroid Carcinoma

**DOI:** 10.1155/2022/4680064

**Published:** 2022-05-14

**Authors:** Liuhua Zhou, Qiaodan Zhu, Jincao Yao, Chen Yang, Dong Xu

**Affiliations:** ^1^Zhejiang Chinese Medical University, Hangzhou, China; ^2^The 2nd Clinical Medical College of Zhejiang Chinese Medical University, Hangzhou, China; ^3^Cancer Hospital of the University of Chinese Academy of Sciences (Zhejiang Cancer Hospital), Hangzhou, China; ^4^Institute of Basic Medicine and Cancer (IBMC), Chinese Academy of Sciences, Hangzhou, China; ^5^Key Laboratory of Head & Neck Cancer Translational Research of Zhejiang Province, Hangzhou, China; ^6^Zhejiang Provincial Research Center for Cancer Intelligent Diagnosis and Molecular Technology, Hangzhou, China

## Abstract

**Background:**

Papillary thyroid carcinoma (PTC) is the most common thyroid carcinoma and is prone to cervical lymph node metastases (CLNM). We aim to analyze the correlation between clinical information, ultrasonic parameters of PTC, and CLNM.

**Methods:**

1335 patients who had pathologically confirmed unifocal PTC were enrolled in this retrospective cohort study. Univariate and multivariate logistic analyses were performed to predict CLNM in PTC patients. The receiver operating characteristic (ROC) curve was used to evaluate the diagnostic performance.

**Results:**

Univariate analysis showed that gender, age, maximum tumor diameter and volume, and cross-sectional and longitudinal aspect ratio were related to CLNM (*P* < 0.05). Multivariate logistic analysis showed that gender, age, maximum tumor diameter, and volume were independent correlative factors, and the cross-sectional aspect ratio had significant difference for PTC2 to predict CLNM. The area under the curve (AUC) of the maximum tumor diameter and volume was 0.738 and 0.733, respectively. Maximum tumor diameter and volume and the cross-sectional and longitudinal aspect ratio were statistically significant following analysis of variance (*P* < 0.05).

**Conclusions:**

Younger age, male, and larger tumor were high risk factors for CLNM in patients with unifocal PTC. The cross-sectional aspect ratio had a more effective predictive value for CLNM in patients with larger thyroid tumors.

## 1. Background

Papillary thyroid carcinoma (PTC) is the most common pathological type of thyroid carcinoma, accounting for approximately 70-80% [1, 2]. Early and timely PTC surgery results in better prognosis; however, PTC is prone to developing cervical lymph node metastases (CLNM), and there is a risk of postoperative recurrence and distant metastases [3]. Although intraoperative lymph node dissection can effectively reduce the residual tumor and recurrence, complications such as recurrent laryngeal nerve injury and hypoparathyroidism can occur. Whether to perform preventive dissection of central neck lymph nodes is still a hot topic of debate among scholars [4]. Therefore, an effective method to increase the detection rate of metastatic cervical lymph nodes before surgery is required.

Ultrasonography (US) is currently the primary method for diagnosing thyroid lesions, but it has some limitations in the detection of CLNM, especially for central lymph node metastases; ultrasonography has the detection rate of 18.8%-31% [5, 6]. US can be affected by interference due to underlying thyroid diseases and surrounding tissues and is dependent on physician experience. Therefore, the identification of useful clues when performing routine thyroid scanning is essential to improve the detection rate of CLNM.

Based on previous studies, the inclusion criteria for samples were being multiplex and multifocal and the aspect ratio (other than cross-sectional or longitudinal), and there was a lack of clear conclusions on tumor volume [7–9]. The present study was based on a stratification which included basic clinical information and US measurements for unifocal PTC, in order to analyze the correlative factors of CLNM. The parameters included were easy to access following basic training in China, especially for thyroid physical examination, and could be the first line of defense to predict the risk of lymph node metastases before surgery when we perform routine thyroid and cervical lymph node ultrasonic screening.

## 2. Materials and Methods

### 2.1. Patients

A total of 3570 patients who underwent thyroidectomy in our hospital from July 2014 to September 2018 were enrolled. The inclusion criteria were as follows: (1) thyroidectomy performed for the first time, (2) enhanced computer tomography (CT) scan of the neck and thorax to assess cervical lymph node and pulmonary metastases and US of the thyroid and neck before surgery, and (3) PTC confirmed by biopsy before surgery. The exclusion criteria were as follows: (1) lung or other distant metastases and (2) multifocal PTC diagnosed by postoperative pathology. Unilateral thyroid lobe plus isthmus excision or total thyroidectomy was performed in these patients. If lateral lymph node metastases were suspected following a comprehensive preoperative evaluation and confirmed by biopsy, lateral lymph node dissection was performed [10]. All patients underwent preventive central lymph node dissection [11, 12], and all lymph nodes were confirmed by pathology. There were two groups, and papillary thyroid microcarcinoma (PTMC) was divided into group PTC1 and group PTC2 which includes PTC above pT1a. This study was approved by the Ethics Committee of the Cancer Hospital of the University of Chinese Academy of Sciences (Zhejiang Cancer Hospital), and all enrolled patients signed an informed consent form.

### 2.2. Instruments

GE Logiq E9 ultrasonographic instrument (General Electric Healthcare, Milwaukee, WI, USA) with a high-resolution linear probe (ML6-15) and Philips iU22 ultrasonographic instrument (Royal Dutch Philips Electronics, Amsterdam, Noord-Holland, Netherlands) with a high frequency linear probe (L12-5) were used for the examination.

### 2.3. Protocol

Patients were maintained in the supine position with the neck hyperextended, and then, the thyroid and both sides of the neck were scanned in multisections. The length, depth, width, location, and features of the tumor were recorded and evaluated from workstations. The ultrasonic images were obtained by the same three professional physicians with more than 10-year experience; all were board-certified physicians with training and experience in thyroid US. The ultrasonic images and reports were analyzed in a blinded manner by two ultrasound specialists (with more than 10-year experience) independently. The imaging data were compared with the pathological results from neck dissections. In cases of discordance, experienced sonographers (with more than 20-year experience) in thyroid US reviewed the images and made the final decision.

Clinical information and US measurements were collected. Clinical information included gender and age. An age threshold of 55 years was used for analysis according to the 8th edition of the United States Joint Committee on Cancer, as the diagnostic age of the TNM staging system for thyroid cancer was 55 years [13]. US measurements included the maximum tumor diameter, tumor volume, cross-sectional aspect ratio, and longitudinal aspect ratio.

The three diameters of the tumor were stated precisely as follows: we did a longitudinal scan of the thyroid, selected the maximum section of the nodule, measured the maximum long diameter (length), and then measured the vertical diameter of the long diameter (height). A transversal scan of the thyroid was executed, the maximum section of the nodule was selected, and the maximum diameter from left to right (width) was measured. The maximum diameter was the maximum of length, width, and height, volume = 0.523∗length∗width∗height, cross‐sectional aspect ratio = height/width, and longitudinal aspect ratio = height/length.

### 2.4. Statistical Analysis

The obtained data were statistically analyzed by SPSS 20.0 software. Continuous quantitative data were expressed as the mean ± standard deviation (SD). Data counting was described statistically by the number of cases and rates. The chi-square test and independent-sample *t*-test were used for univariate analysis. A multivariate analysis using binary logistic regression analysis was adopted if analysis index *P* < 0.05 in the univariate analysis.

Odds ratios (ORs) with 95% confidence intervals (CIs) were calculated, and receiver operating characteristic curves (ROC) were analyzed for factors with significance on binary linear regression analysis. Analysis of variance (ANOVA) was used for the positive group. The potential errors due to multiple comparisons for the secondary group and subgroup were handled by adjusting significance threshold. *P* < 0.05 was considered statistically significant.

## 3. Results

A total of 1335 patients with 1335 lesions were included, PTC1 was 874 and PTC2 was 461, and the clinical data and ultrasonic measurements were retrospectively analyzed. There were 299 males and 1036 females, aged 12 to 84 years, with an average age of 45.3 ± 11.8 years; the maximum tumor diameter was 3.5 to 64.7 mm, with an average diameter of 10.3 ± 7.9 mm. 432 cases were in the positive group (32.4%): PTC1 had 181 cases and PTC2 had 251 cases. Among the positive group, 285 (66.0%) with only central lymph node metastases, 54 (12.5%) with only lateral lymph node metastases, and 93 (21.5%) with metastases both in central and lateral lymph nodes were included. There were 903 cases included in the negative group (67.6%): PTC1 had 693 cases and PTC2 had 210 cases. 143 patients underwent lateral lymph node dissection due to the positive preoperative biopsy. Lateral neck lymph node dissection was performed in 5 cases with high imaging suspicion but negative puncture results. Three cases of them had lateral neck lymph node metastases, and 2 cases were confirmed negative during operation.

By comparing the positive and negative group, the results showed that males (*x*^2^ = 18.011, *P* < 0.001), age (*t* = 9.132, *P* < 0.001), age < 55 years (*x*^2^ = 20.599, *P* < 0.001), maximum tumor diameter (*t* = −13.922, *P* < 0.001) and volume (*t* = −7.927, *P* < 0.001), cross-sectional aspect ratio (*t* = 3.895, *P* < 0.001), and longitudinal aspect ratio (*t* = 5.721, *P* < 0.001) were all significantly related to CLNM (Table 1).

We divided two subgroups in the positive group: there were PTC1 and PTC2. By comparing these two groups, the cross-sectional aspect ratio ≥ 1 had significant difference for PTC2 to predict CLNM (Table 2).

**Figure 1:**
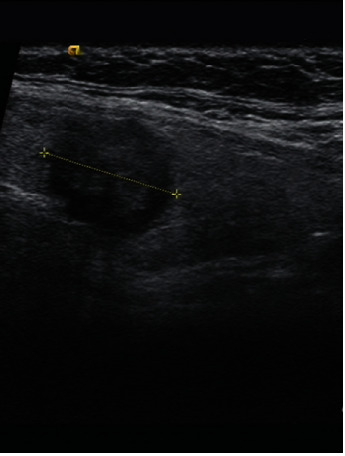
Male 50 years old, left thyroid papillary carcinoma, size 15.5∗13∗13.2 mm, central lymph node metastases.

**Figure 2:**
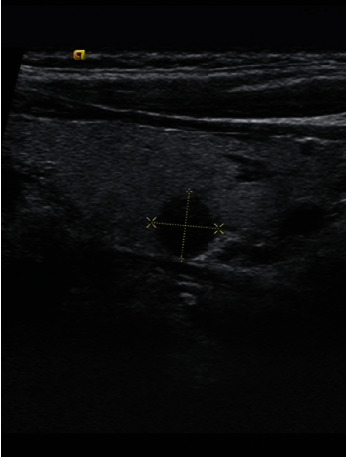
Female 42 years old, left thyroid papillary microcarcinoma, size 7.5∗7∗5.7 mm, no cervical lymph node metastases.

Gender, age, maximum tumor diameter and volume, and cross-sectional and longitudinal aspect ratios were all included in the logistical analysis. The results showed that males (OR = 1.723, 95% CI 1.281-2.317, *P* < 0.001), age (OR = 0.960, 95% CI 0.949-0.971, *P* < 0.001), maximum tumor diameter (OR = 1.188, 95% CI 1.148-1.230, *P* < 0.001), and volume (OR = 0.838, 95% CI 0.780-0.902, *P* < 0.001) were independent correlative factors for CLNM (Table 3, Figures 1 and 2).

Logistic regression analysis was carried out to determine independent correlative factors of CLNM. Gender, age, maximum tumor diameter, and volume were analyzed using the ROC curve. The AUC, specificity, and sensitivity of the maximum tumor diameter were 0.738, 66.6%, and 69.9%, respectively; the AUC, specificity, and sensitivity of the tumor volume were 0.733, 67.6%, and 68.1%, respectively (Table 4).

According to the differences in clinical and ultrasonic characteristics using ANOVA, the patients were divided into three pair-to-pair comparison groups: only central metastasis group, only lateral metastasis group, and both metastasis group. The results showed that the significant variable (*P* < 0.05) between the only central and only lateral metastasis groups and the only central and both metastasis group was the maximum tumor diameter. The significant variables (*P* < 0.001) between the only central and both metastasis group included tumor volume, cross-sectional aspect ratio, and longitudinal aspect ratio. The comparison between the three pair-to-pair groups is shown in Table 5.

## 4. Discussion

Thyroid carcinoma is a common endocrine malignant carcinoma, and PTC has the highest incidence of all thyroid carcinomas. PTC shows low malignant growth with a low incidence of distant metastases and a low mortality rate; however, CLNM tends to occur at the early stage [14]. Lymph node metastases in PTC are related to the diameter, location, number, and invasive growth of the primary tumor [14, 15]. There is no uniform conclusion on the correlation between gender, age, pathological type, and lymph node metastases [16, 17]. Ultrasonography is the primary examination method for the thyroid gland, but the detection of cervical lymph nodes by US is 18.8%-31% and is limited due to interference from the trachea, esophagus, and osseous tissue, underlying thyroid diseases, and the examiner's experience [5, 18]. Therefore, evaluation of the correlative factors of CLNM in PTC has great clinical value. Previous similar studies have not provided consistent conclusions, as some included fewer cases, some included multifocality, and some included complex parameter characteristics. This study only enrolled unifocal PTC and analyzed the clinical information and US measurements. A stratified study is necessary, with additional subsequent and multiple ultrasonic signs, TI-RADS (Thyroid Imaging, Reporting and Data System), multifocality, pathological types, and so on, in order to obtain more complete and systematic research results.

PTC is more common in female patients, with a male to female ratio of approximately 1 : 3, and the ratio in this study was 1 : 3.46. Mao et al. [19] and Heaton et al. [20] reported that women and elderly patients were at risk of PTC, while men and younger patients were at risk of CLNM. Sun et al. [21] confirmed that men had an increased risk of central neck lymph node metastases. In this study, CLNM occurred in 42.5% (127/299) of male patients and occurred in only 29.4% (305/1036) of female patients, which also suggests that male patients have a higher risk of lymph node metastases. This is consistent with results reported in the literature.

PTC occurs in all age ranges, with a high incidence between the age of 30 and 60 years. The mean age in the positive group included in this study was younger than that in the negative group, and the difference was statistically significant (*P* < 0.001), which was also consistent with previous reports where age was an independent risk factor for CLNM in PTC [6, 22]. We used 55 years as the threshold in this study according to the TNM staging system for thyroid cancer [13], and 35.4% (371/1048) of patients who were younger than 55 years had CLNM, and among those who were 55 years or older, lymph node metastases occurred in only 21.2% (61/287) of patients in this study. This was consistent with the study reported by Zhou et al. [4], where age was an independent risk factor for CLNM, and the risk of CLNM in patients who were younger than 55 years was 2.6 times that in patients who were 55 years or older.

The maximum tumor diameter is an important reference index for PTC treatment protocols and the range of surgery [8]. The diameter of the lesion was closely related to invasion of the tumor. The growth of a malignant tumor is a process of self-proliferation and external invasion, the range of invasion continues to expand, and the contact area between the cancer focus and the capsule, blood vessels, and lymphatic vessels of the thyroid also increases [21]; thereby, the incidence of lymph node metastases also increases [23]. A retrospective analysis based on large samples has shown that CLNM tended to occur in patients with a maximum tumor diameter of 10 mm or larger [24, 25]. The results demonstrated that the maximum tumor diameter in the positive group was about 1.7 times that in the negative group. The tumor seemed to be ellipsoid, and the volume as the evaluation parameter made the result more objective and scientific. The tumor volume in the positive group was about 3.5 times that in the negative group. Tumor volume was significantly different between the only central metastasis group and the central and lateral metastasis group (*P* < 0.001). For larger tumors, cervical lymph nodes should be examined in order to improve the detection rate of CLNM. In particular, in patients with large volume tumors, central or both central and lateral lymph node metastases should be determined in advance. If both of these examinations were added to routine thyroid and cervical lymph node ultrasonic screening, it would provide a guide for performing FNA for the detection of CLNM before surgery.

An aspect ratio ≥ 1 is a highly specific index for the diagnosis of malignant thyroid nodules [26, 27]. Zhan and Xu [28] showed that the sensitivity of the aspect ratio in the differential diagnosis of benign and malignant thyroid nodules decreased gradually as the volume of thyroid nodules increased, and an aspect ratio ≥ 1 was more significant for the diagnosis of PTC with a smaller volume. Nam et al. [29] divided 488 cases of PTC into two groups with one group having malignant ultrasonic signs, including an aspect ratio > 1, solid mass with low echo, microcalcification, and a blurred boundary, while the other group had no malignant ultrasonic signs. A comparison between these two groups showed that patients with PTC and malignant signs were more prone to CLNM. Studies by Zhou et al. [4] showed that an aspect ratio > 1 in 1174 cases of unifocal PTC was a risk factor for CLNM. Deng et al. [30] reported that no statistical significance was seen in 908 PTC patients with an aspect ratio ≥ 1 in predicting cervical lateral lymph node metastases. Combined with the previous literature, on the one hand, there was no distinction between the transverse or longitudinal aspect ratio; on the other hand, the correlation between the aspect ratio and PTC CLNM was inconsistent.

According to the morphology of the tumor, univariate analysis demonstrated that the cross-sectional aspect ratio and longitudinal section aspect ratio were both statistically significant (*P* < 0.001) in this study, while logistic regression analysis showed that there was no statistically significant difference between the two groups. The main reason was that PTMC patients were 65.5% (874/1335) in the study; just 20.7% (181/874) were in the positive group. We compared the cross-sectional and longitudinal section aspect ratio for PTC1 and PTC2; it could be concluded that the cross-sectional aspect ratio had a better predictive value for CLNM in PTC2, compared with the longitudinal aspect ratio. There was less relevant literature with the association between cross-sectional and longitudinal section aspect ratios in predicting cervical LN metastases, especially for PTC2. We will collect more data for further research in the future.

US is the preferred choice for imaging diagnosis and will become the first line of defense for the detection of cervical metastatic lymph nodes. However, neck CT and magnetic resonance imaging (MRI) are also common imaging methods; their advantage is to find the retropharyngeal, deep tracheoesophageal groove and upper mediastinal lymph nodes that are difficult to be detected by US. For patients including extranodal invasion, large lymph node, or multiple lymph node metastases, CT and MRI can acquire the relationship between the lesion and the surrounding tissue, both of them are easier to clarify the distribution of lymph nodes [31]. We can combine with appropriate imaging methods for more accurate diagnostic results and help the operator make preoperative planning.

The limitations of this study were as follows. (1) This was a single-center retrospective study including unifocal PTC and lymph node dissection performed in the central area, which may have introduced subjective bias. (2) Cases with metastases in the lateral location were not adequate, and large samples are required to study the cervical metastases in different parts. For skip lateral lymph node metastases, more effective preoperative assessment should be adopted. (3) This is the preliminary study for large sample size of PTC patients. In further research, we will add detailed TI-RADS, clinical and pathological staging, subdivided pathological types, and machine learning models.

## 5. Conclusions

In summary, based on PTC pathology, the correlations between gender, age, US measurements, and CLNM were analyzed in order to assess the risk of lymph node metastases before surgery. For PTC patients with risk factors such as younger age, male gender, and larger tumor, more detailed preoperative lymph node examination should be conducted, including a cervical lymph node sonographic scan by experienced sonographers (with more than 10-year experience) and CT scan of the neck as a rule, which will cover a wide range of thyroid cancer population and help develop a more reasonable clinical treatment protocol.

## Figures and Tables

**Table 1:** Univariate analysis of correlative factors of CLNM in PTC.

Variable	Positive group (*n* = 432)	Negative group (*n* = 903)	Statistics	*P*
Gender (male/female)	127/305	172/731	*x* ^2^ = 18.011	*P* < 0.001
Age	41.20 ± 12.20	47.30 ± 11.02	*t* = 9.132	*P* < 0.001
Age (≥55years/<55years)	61/371	226/677	*x* ^2^ = 20.599	*P* < 0.001
Tumor maximum diameter (mm)	14.4 ± 10.0	8.4 ± 5.7	*t* = −13.922	*P* < 0.001
Tumor volume (ml)	2.1 ± 4.6	0.5 ± 2.4	*t* = −7.927	*P* < 0.001
Cross-sectional aspect ratio	1.02 ± 0.28	1.08 ± 0.26	*t* = 3.895	*P* < 0.001
Longitudinal aspect ratio	0.88 ± 0.29	0.98 ± 0.31	*t* = 5.721	*P* < 0.001

**Table 2:** Univariate analysis of PTC1 and PTC2 in the positive group.

Variable	Cross-sectional aspect ratio ≥ 1	Longitudinal section aspect ratio ≥ 1	*P*
			*P* = 0.006
PTC1+CLNM (181)	118	93	
PTC2+CLNM (251)	80	32	

**Table 3:** Logistic regression analysis of correlative factors of CLNM in PTC.

Variable	OR	95% CI	*P*
Gender (male/female)	1.723	1.281~2.317	*P* < 0.001
Age	0.960	0.949~0.971	*P* < 0.001
Tumor maximum diameter	1.188	1.148~1.230	*P* < 0.001
Tumor volume	0.838	0.780~0.902	*P* < 0.001
Cross-sectional aspect ratio	1.166	0.625~2.173	*P* = 0.629
Longitudinal aspect ratio	1.313	0.751~2.298	*P* = 0.340

**Table 4:** AUC and cut-off values of correlative factors for CLNM in PTC.

Variable	AUC	Specificity	Sensitivity	Cut-off value
Gender (male/female)	0.552	81.0%	29.4%	Not applicable
Age	0.355	99.9%	0.2%	80
Tumor maximum diameter	0.738	66.6%	69.9%	8.4 mm
Tumor volume	0.733	67.6%	68.1%	0.2 ml

**Table 5:** ANOVA variance analysis for CLNM in PTC.

Variable	Central vs. lateral	Central vs. both	Lateral vs. both
Gender (male/female)	*P* = 1.000	*P* = 0.677	*P* = 0.912
Age	*P* = 0.725	*P* = 0.743	*P* = 0.272
Tumor maximum diameter	*P* = 0.002^a^	*P* < 0.001^a^	*P* = 0.270
Tumor volume	*P* = 0.121	*P* < 0.001^a^	*P* = 0.987
Cross-sectional aspect ratio	*P* = 0.431	*P* < 0.001^a^	*P* = 0.768
Longitudinal aspect ratio	*P* = 0.124	*P* < 0.001^a^	*P* = 0.104

^a^ *P* < 0.05.

## Data Availability

The scientific guarantor of this publication is Professor Dong Xu. Permission for any material in the article to be used can be obtained from all authors.

## Abbreviations

PTC: Papillary thyroid carcinoma
CLNM: Cervical lymph node metastases
PTMC: Papillary thyroid microcarcinoma
FNA: Fine-needle aspiration biopsy
US: Ultrasonography
ROC: Receiver operating characteristic
OR: Odds ratio
CI: Confidence interval
AUC: Area under the curve
CT: Computer tomography
MRI: Magnetic resonance imaging.
